# High enhancer activity is an epigenetic feature of HPV negative atypical head and neck squamous cell carcinoma

**DOI:** 10.3389/fcell.2022.936168

**Published:** 2022-07-19

**Authors:** S. Carson Callahan, Veena Kochat, Zhiyi Liu, Ayush T. Raman, Margarita Divenko, Jonathan Schulz, Christopher J. Terranova, Archit K. Ghosh, Ming Tang, Faye M. Johnson, Jing Wang, Heath D Skinner, Curtis R. Pickering, Jeffrey N. Myers, Kunal Rai

**Affiliations:** ^1^ Department of Genomic Medicine, University of Texas MD Anderson Cancer Center, Houston, TX, United States; ^2^ Department of Head and Neck Surgery, The University of Texas MD Anderson Cancer Center, Houston, TX, United States; ^3^ Department of Surgical Oncology, University of Texas MD Anderson Cancer Center, Houston, TX, United States; ^4^ Graduate Program in Quantitative Sciences, Baylor College of Medicine, Houston, TX, United States; ^5^ Epigenomics Program, Broad Institute of MIT and Harvard, Cambridge, MA, United States; ^6^ Department of Data Science, Dana-Farber Cancer Institute, Boston, MA, United States; ^7^ The University of Texas Graduate School of Biomedical Sciences, Houston, TX, United States; ^8^ Department of Thoracic/Head & Neck Medical Oncology, The University of Texas MD Anderson Cancer Center, Houston, TX, United States; ^9^ Department of Bioinformatics and Computational Biology, The University of Texas MD Anderson Cancer Center, Houston, TX, United States; ^10^ Department of Radiation Oncology, The University of Texas MD Anderson Cancer Center, Houston, TX, United States; ^11^ Department of Radiation Oncology, University of Pittsburgh, UPMC Hillman Cancer Center, Pittsburgh, PA, United States

**Keywords:** epigenome analyses, head and neck cancer, enhancer regulation, BET inhibitors, drug resistance

## Abstract

Head and neck squamous cell carcinoma (HNSCC) is a heterogeneous disease with significant mortality and frequent recurrence. Prior efforts to transcriptionally classify HNSCC into groups of varying prognoses have identified four accepted molecular subtypes of the disease: Atypical (AT), Basal (BA), Classical (CL), and Mesenchymal (MS). Here, we investigate the active enhancer landscapes of these subtypes using representative HNSCC cell lines and identify samples belonging to the AT subtype as having increased enhancer activity compared to the other 3 HNSCC subtypes. Cell lines belonging to the AT subtype are more resistant to enhancer-blocking bromodomain inhibitors (BETi). Examination of nascent transcripts reveals that both AT TCGA tumors and cell lines express higher levels of enhancer RNA (eRNA) transcripts for enhancers controlling BETi resistance pathways, such as lipid metabolism and MAPK signaling. Additionally, investigation of higher-order chromatin structure suggests more enhancer-promoter (E-P) contacts in the AT subtype, including on genes identified in the eRNA analysis. Consistently, known BETi resistance pathways are upregulated upon exposure to these inhibitors. Together, our results identify that the AT subtype of HNSCC is associated with higher enhancer activity, resistance to enhancer blockade, and increased signaling through pathways that could serve as future targets for sensitizing HNSCC to BET inhibition.

## Introduction

Head and neck squamous cell carcinoma (HNSCC) is the sixth most common cancer worldwide and the predominant form of head and neck cancer (Marur et al., 2010; Zhou et al., 2016). In the United States, over 60,000 new HNSCC cases and more than 13,000 HNSCC deaths are reported per year (Zhou et al., 2016). HNSCC covers a wide variety of anatomical sites, including the oral cavity, oropharynx, hypopharynx, and larynx (Forastiere et al., 2001). The prognosis for HNSCC is overall poor with a 5-year survival of approximately 50%, which has remained relatively unchanged for decades (Marur et al., 2010). This is largely attributed to factors such as late stage at initial presentation and high rates of primary tumor recurrence (Bonner et al., 2006; Pickering et al., 2013). Treatment for HNSCC involves combinations of surgery, chemotherapy, and radiotherapy, with exact treatment plans depending on tumor location and TNM stage (Marur et al., 2010; Zhou et al., 2016).

To date, studies on HNSCC have focused largely on genomic characterizations such as exome sequencing and copy number alterations. The most common alterations, such as mutations in *TP53* at 17p13 and alterations in *p16* at 9p21, have been known for decades (Forastiere et al., 2001; Zhou et al., 2016). More recent comprehensive analyses of HNSCC tumors have supported these previous findings, in addition to identifying common alterations in the Notch1 pathway and cell cycle genes (Agrawal et al., 2011; Pickering et al., 2013; Cancer Genome Atlas, 2015). Unfortunately, very few of these studies have resulted in clinically actionable findings. There are, however, some disputed exceptions, such as the EGFR inhibitor cetuximab, which showed benefit when combined with radiotherapy (Bonner et al., 2006).

One interesting result of these and other studies is the notion of molecular subtypes of disease. Inspired by similar studies in other tumors such as breast, lung, and brain, transcriptomic data from patient samples was used to classify head and neck tumors into 4 subtypes: Atypical (AT), Basal (BA), Classical (CL), and Mesenchymal (MS) (Chung et al., 2004; Walter et al., 2013; Cancer Genome Atlas, 2015). These studies have largely focused on the relationship of these subtypes to genomic alterations, such as mutation patterns, copy number changes, or alterations in key transcription factor expression, and clinical features, such as progression free survival and lymph node metastasis at time of diagnosis. However, there have been very few studies describing the epigenomic features of the subtypes. The importance of chromatin modification states in HNSCC is further evidenced by the finding that global levels of certain histone tail modifications correlate with clinical measurements such as tumor stage, cancer-specific survival, and disease-free survival in oral squamous cell carcinoma (Chen et al., 2013). Because there are currently only a sparse number of HNSCC epigenomics datasets, particularly in the realm of histone modifications and chromatin regulation, there remains an unmet need to investigate these aspects of gene regulation and leverage newly discovered biology to better define the disease and develop new therapeutic approaches (Castilho et al., 2017; Serafini et al., 2020).

Since HNSCC subtypes are defined by their transcriptomic signatures, it stands to reason they would also have unique epigenomic features, such as enhancer landscapes, that may, in part, be driving the defining transcriptomic signatures. Through mapping of H3K27ac-marked active enhancers in 28 HPV-negative HNSCC cell lines, we demonstrate that the AT subtype is characterized by high enhancer activity. Consistently, the AT subtype was associated with resistance to enhancer-blocking bromodomain inhibitors (BETi). BETi resistance pathways specifically showed high enhancer activity as measured by nascent enhancer RNAs (eRNAs) and enhancer-promoter contacts, providing mechanistic insights into the aggressive nature of the AT subtype. Overall, our data suggests high enhancer activity as an epigenetic feature of atypical HNSCCs.

## Methods

### Cell culture

Human HNSCC cell lines were acquired and characterized as previously described (Zhao et al., 2011). Briefly, cell lines were cultured in DMEM supplemented with 10% FBS, L-glutamine, sodium pyruvate, nonessential amino acids, vitamins, and 1% penicillin-streptomycin. All cell lines were cultured at 37°C in an atmosphere of 5% CO_2_.

### RNA-Sequencing processing and analysis

RNA-seq data for cell line subtype assignments were obtained as raw counts, processed as previously described (data available at GEO accession GSE122512) (Kalu et al., 2017; Gleber-Netto et al., 2019). To assign HNSCC cell lines to their representative subtypes, we used the HNSCC gene list templates generated by Yu et al. (Yu et al., 2019) and utilized the CMScaller workflow and implementation of the NTP algorithm to find the closest matching subtype for each cell line (FDR <0.1) based on their transcriptomic profiles (Hoshida, 2010; Eide et al., 2017). Upregulated genes for each subtype were computed using CMScaller in a one-vs-rest fashion.

For the PLX51107 treatment RNA-seq experiments, representative cell lines were selected for the AT subtype (HN4) and a non-AT subtype (MDA1186, CL subtype) and treated with DMSO, GR_50_ of the MDA1186, or GR_50_ of HN4 for 24 h prior to RNA isolation. RNA extraction was performed using an RNeasy Mini Kit per manufacturer’s instructions (Qiagen). Isolation of mRNA was performed using NEBNext Poly(A) mRNA Magnetic Isolation Module and libraries were prepared using the NEBNext Ultra II Directional RNA Library Prep kit (New England BioLabs). Library quality was checked on an Agilent TapeStation 4150 and quantified by Qubit 2000 fluorometer (Invitrogen). Libraries were pooled in equimolar ratios and sequenced on Illumina NovaSeq6000 SP runs with paired-end 100-bp reads at The Advanced Technology Genomics Core (ATGC) at MD Anderson Cancer Center.

PLX51107 treatment RNA-seq raw reads were processed using the provided pipeline: https://github.com/sccallahan/QUACKERS_RNAseq-pipeline. In brief, raw reads were aligned to the hg19 genome using STAR v2.7.2b (Dobin et al., 2013) and quality checked using FastQC v0.11.8 (https://www.bioinformatics.babraham.ac.uk/projects/fastqc/). Counts were generated using featureCounts from subread v1.6.3 80 (Liao et al., 2013). Downstream normalization and differential expression analysis were performed using DESeq2, with size factors being calculated using data-driven housekeeping gene method as implemented in the CustomSelection R package (Love et al., 2014; Dos Santos et al., 2020). Pathway enrichment analyses were performed using GSEA’s pre-ranked list option (Subramanian et al., 2005). Overlaps of HN4 and MDA1186 low dose PLX51107 differentially expressed genes were performed using the VennDiagram package in R, and the HN4 uniquely upregulated gene list was subjected to pathway enrichment analysis using the gsea-msigdb online tool (http://www.gsea-msigdb.org/gsea/msigdb/annotate.jsp).

CCLE RNA-seq data were downloaded as raw counts from the DepMap download portal (https://depmap.org/portal/download/). Subtype assignment and downstream analysis were carried out as above.

### Whole exome sequencing processing and analysis

Whole exome sequencing (WES) data was processed as previously described and obtained as a MAF file from the authors (Kalu et al., 2017; Gleber-Netto et al., 2019). To cluster the cell lines based on mutation background, all mutation calls were binarized to 1 or 0 to represent “mutated” or “not mutated,” respectively. The Jaccard distance matrix was then computed, and the resulting matrix was clustered using Ward’s minimum variance method. Total mutational burden was calculated by summing the number of mutations per sample, then grouping the samples based on their assigned molecular subtype. Data for cell line tissue of origin and “source” were obtained as previously described (Zhao et al., 2011).

### ChIP-Sequencing processing and analysis

ChIP assays were performed as described previously (Terranova et al., 2018). Briefly, approximately 2 × 10^7^ cells were harvested by scraping. Samples were cross-linked with 1% (wt/vol) formaldehyde for 10 min at 37°C with shaking. After quenching with 150 mM glycine for 5 min at 37°C with shaking, cells were washed twice with ice-cold PBS and frozen at −80°C for further processing. Cross-linked pellets were thawed and lysed on ice for 30 min in ChIP harvest buffer (12 mM Tris-Cl, 1 × PBS, 6 mM EDTA, 0.5% SDS) with protease inhibitors (Sigma). Lysed cells were sonicated with a Bioruptor (Diagenode) to obtain chromatin fragments (∼200–500 bp) and centrifuged at 15,000 × g for 15 min to obtain a soluble chromatin fraction. In parallel with cellular lysis and sonication, antibodies (5 μg/3 × 10^6^ cells) were coupled with 30 μL of magnetic protein G beads in binding/blocking buffer (PBS +0.1% Tween +0.2% BSA) for 2 h at 4°C with rotation. The antibody used for ChIP was anti-H3K27ac (Abcam; ab4729). Soluble chromatin was diluted five times using ChIP dilution buffer (10 mM Tris-Cl, 140 mM NaCl, 0.1% DOC, 1% Triton X, 1 mM EDTA) with protease inhibitors and added to the antibody-coupled beads with rotation at 4°C overnight. After washing, samples were treated with elution buffer (10 mM Tris-Cl, pH 8.0, 5 mM EDTA, 300 mM NaCl, 0.5% SDS), RNase A, and Proteinase K, and cross-links were reversed overnight at 37°C. Immune complexes were then washed five times with cold RIPA buffer (10 mM Tris–HCl, pH 8.0, 1 mM EDTA, pH 8.0, 140 mM NaCl, 1% Triton X-100, 0.1% SDS, 0.1% DOC), twice with cold high-salt RIPA buffer (10 mM Tris–HCl, pH 8.0, 1 mM EDTA, pH 8.0, 500 mM NaCl, 1% Triton X-100, 0.1% SDS, 0.1% DOC), and twice with cold LiCl buffer (10 mM Tris–HCl, pH 8.0, 1 mM EDTA, pH 8.0, 250 mM LiCl, 0.5% NP-40, 0.5% DOC). ChIP DNA was purified using SPRI beads (Beckman Coulter) and quantified using the Qubit 2000 (Invitrogen) and TapeStation 4150 (Agilent). Libraries for Illumina sequencing were generated following the New England BioLabs (NEB) Next Ultra DNA Library Prep Kit protocol. Amplified ChIP DNA was purified using double-sided SPRI to retain fragments (∼200–500 bp) and quantified using the Qubit 2000 and TapeStation 4150 before multiplexing.

Raw fastq reads for all ChIP-seq experiments were processed using a Snakemake based pipeline https://github.com/crazyhottommy/pyflow-ChIPseq. Briefly, raw reads were first processed using FastQC and uniquely mapped reads were aligned to the hg19 reference genome using Bowtie version 1.1.2 (Langmead et al., 2009). Duplicate reads were removed using SAMBLASTER (Faust and Hall, 2014) before compression to bam files. To directly compare ChIP-seq samples, uniquely mapped reads for each mark were downsampled per condition to 15 million, sorted, and indexed using samtools version 1.5 (Li et al., 2009). To visualize ChIP-seq libraries on the IGV genome browser, we used deepTools version 2.4.0 (Ramirez et al., 2016) to generate bigWig files by scaling the bam files to reads per kilobase per million (RPKM) and WiggleTools (Zerbino et al., 2014) to create average profile plots for each molecular subtype.

Peak overlaps were performed by first generating consensus peak files for each subtype, defined as any peak found in at least 2 samples from the subtype. The resulting 4 peaksets (one per subtype) were then used as input for intervene’s (Khan and Mathelier, 2017) upset module to generate upset plots and common/unique peaksets for further analysis. Gene linkage was performed using previously published enhancer-promoter linkage data from Cao et al. (Cao et al., 2017), and the resulting gene list was used as input for pathway enrichment analysis using the gsea-msigdb online tool. Enrichment plots were generated using two definitions of common peaks. The first method uses DiffBind (Stark and Brown, 2020) (https://bioconductor.org/packages/release/bioc/html/DiffBind.html) to define a peakset using any peak found in at least 2 samples, irrespective of subtype. The second uses the peaks found in all subtypes when overlapped using the intervene package mentioned previously. Both resulting peaksets were used as input for ngs.plot (Shen et al., 2014) to generate figures.

### Drug response assays

Cell confluence and proliferation were measured using the IncuCyte ZOOM system (Essen Biosciences). For each cell line, seeding density was optimized such that the cells would be in their exponential growth phase for the duration of drug treatment. On day 0, cells were seeded into 96 well plates and left in the incubator overnight. On day 1, media containing either drug (PLX51107 or OTX015) or DMSO was added to the wells. Plates were then left in the IncuCyte ZOOM with treated media for 72 h, at which point cell confluence was measured. Each assay was performed in biological duplicate with technical triplicate wells. Drug response metrics were calculated using GR metrics (Hafner et al., 2016) using cell confluence as a proxy for growth rate, and GR_AOC_ values from each cell line were combined based on their molecular subtype for statistical analysis.

CCLE drug response data were downloaded and processed using the PharmacoGx “auc_recomputed” dataset (Smirnov et al., 2016). Compounds which were missing data for more than 25% of samples were excluded. For the JQ1 analysis, 1 sample was missing data and was imputed using predictive mean matching [as implemented in the mice package (Buuren and Groothuis-Oudshoorn, 2011) (https://www.jstatsoft.org/article/view/v045i03)] on the complete, filtered drug response matrix.

### PRO-Seq processing and eRNA analysis

Extraction of nuclei and precision run-on reaction was carried out as described previously (Mahat et al., 2016). Nuclei were isolated from approximately 10 million cells after treating with 12 ml of ice-cold swelling buffer (10 mM Tris-HCl pH 7.5, 2 mM MgCl2, 3 mM CaCl2) for 10 min and scraping out the cells. After spinning at 600 × g for 10 min at 4°C, the supernatant was removed and the cells were lysed in 10 ml of lysis buffer (10 mM Tris-HCl pH 7.5, 2 mM MgCl2, 3 mM CaCl2, 10% glycerol, 0.5% NP-40, 4 U/ml SUPERase inhibitor) on ice for 5 min. The lysate was spun at 600 × g for 8 min and the nuclei were collected. The nuclei were then resuspended in 1 ml of freezing buffer (50 mM Tris-HCl pH 8.0, 5 mM MgCl2, 0.1 mM EDTA, 40% glycerol) and spun at 900 × g for 10 min. For performing precision nuclear run-on reaction, nuclei were resuspended in 100 µL of freezing buffer and added to 100 µL of NRO-reaction mix - NRO-reaction buffer (10 mM Tris-HCl pH 8.0, 5 mM MgCl2, 300 mM KCl), 1 mM DTT, 100 U/ml SUPERase-In, 1% Sarkosyl, 250 M ATP, 250 M GTP, 50 M biotin-11-UTP, 50 M biotin-11-CTP. Reaction was carried out at 29°C for 4 min. RNA was extracted using TRIzol. Base hydrolysis was carried out by heat denaturing briefly at 65°C for 40 s following by cooling on ice and treatment with 1N NaOH for 6 min on ice. The sample with fragmented RNA was neutralized with 1 M Tris-HCl pH 6.8 and isolated by passing through P-30 column (Biorad, #732-6250). The NRO-reaction products containing biotinylated RNA was purified using Streptavidin C1 beads which were washed thrice with wash buffer (0.1 N NaOH, 50 mM NaCl) and twice with 100 mM NaCl. The washed beads were resuspended in binding buffer (10 mM Tris-HCl pH 7.4, 150 mM NaCl, 0.1% Triton X-100) and added to the sample and incubated at room temperature for 30 min on a rotator. After removing the supernatant using a magnetic stand, beads were washed twice with high salt wash buffer (50 mM Tris-HCl pH 7.4, 2 M NaCl, 0.5% Triton X-100), once with low salt wash buffer (10 mM Tris-HCl pH 7.4, 300 mM NaCl, 0.1% Triton X-100) and twice with no salt wash buffer (5 mM Tris-HCl pH 7.4, 0.1% Triton X-100). Beads were then resuspended in TRIzol and RNA was extracted. The bead purification of biotinylated RNA was performed once more, and RNA was extracted using TRIzol to improve the purity of the sample.

Libraries were generated based on previously described protocol (Van Nostrand et al., 2016). Isolated RNA samples were dephosphorylated by FastAP (ThermoFisher) and T4 Polynucleotide kinase (NEB). Samples were cleaned up using MyONE Silane beads and RNA was isolated with RLT buffer (Qiagen). To the eluted RNA, a barcoded RNA adapter (RiL19) was ligated to the 3′ end using T4 RNA ligase (NEB). The 3′ adaptor ligated RNA was again cleaned up as mentioned above. RNA was then reverse transcribed with AR17 primer and AffinityScript reverse transcriptase (Agilent). cDNA was then cleaned up by treating with ExoSAP-IT (Affymetrix) to remove excess oligonucleotides. Excess RNA was removed from cDNA by treating with 1 M NaOH at 70°C for 12 min and neutralizing with 1 M HCl. cDNA was then cleaned up with MyONE Silane beads and RLT buffer and eluted in 5 mM Tris-Cl, pH 7.5. A second 5′adaptor (rand3Tr3) was ligated to cDNA with T4 RNA ligase in an overnight reaction at room temperature. The adaptor ligated cDNA was then cleaned up with MyONE Silane beads and RLT buffer and eluted in 10 mM Tris-Cl, pH 7.5. cDNA samples were then PCR amplified using NEBNext^®^ Ultra™ II Q5^®^ Master Mix multiplexing was done with D50X and D70X primers. Libraries were size selected and purified using SPRI beads. Final libraries were quantified using D1000 tapestation and Qubit™ dsDNA HS Assay Kit (Thermo Fisher Scientific) and sequenced using NovaSeq6000 with 100 nt paired-end format.

Fastq files from precision nuclear run-on sequencing (PRO-seq) experiments were processed using the previously described PEPPRO pipeline (Smith et al., 2021). Briefly, fastq files first undergo pre-processing steps of adapter removal, read deduplication, read trimming, and reverse complementation. The resulting files are then “pre-aligned” to the human rDNA genome to siphon off these unwanted reads. The rDNA-removed files are then aligned to the human hg19 genome using bowtie2 (Langmead and Salzberg, 2012). After quality control assessment, 5 samples from the AT subtype (representing the 3 unique cell lines used in the drug response assays) and 3 samples from the CL subtype (representing the 2 unique cell lines used in the drug response assays) were carried forward for further analysis. The aligned, sorted bam files for these samples were used as input for downstream analysis using the previously described NRSA downstream analysis pipeline (Wang et al., 2018). In brief, NRSA uses bidirectional transcription in intergenic regions to identify and call enhancers/eRNAs. A raw counts table for these eRNAs is then generated and fed into the DESeq2 tool for differential expression analysis. Identified enhancers are assigned to their nearest genes to generate a list of genes with upregulated eRNA expression in the AT subtype, which was then used as input for pathway enrichment analysis.

TCGA RPKM expression levels of numerous eRNAs from typical enhancers were downloaded from publicly available datasets (https://bioinformatics.mdanderson.org/Supplements/Super_Enhancer/TCEA_website/parts/3_eRNA_quantification.html) based on previously published work (Chen et al., 2018; Chen and Liang, 2020). TCGA mRNA-seq for subtype assignment was downloaded from FireBrowse (http://firebrowse.org/). TCGA samples were assigned to molecular subtypes by first generating templates based on previously assigned molecular subtypes from the initial HNSCC TCGA cohort (Cancer Genome Atlas, 2015). These assigned subtypes were then expanded to the current cohort of samples by using the CMSCaller functionality described above. As before, samples were only retained for further analysis if they possessed an assignment FDR <0.1. Samples were then grouped into “Atypical” or “Other” based on their molecular subtype, and significant differential expression of eRNAs was determined by > 1.5 fold-change in expression and FDR <0.05. As with the PRO-seq data, these eRNAs were linked to their nearest gene using bedtools *via* the bedr R package (Quinlan and Hall, 2010), and the resulting gene list was used as input for pathway enrichment analysis. Intersections of the TCGA eRNA enriched pathways and PRO-seq eRNA enriched pathways were performed using the Venn Diagram package in R and significance was calculated using the hypergeometric overlap method, with a Universe size set to the number of unique pathways in a particular gene set. Only pathways with a p.adjust <0.25 with a maximum of 20 enriched pathways per gene set were included in the analysis.

### HiChIP protocol, processing, and analysis

HiChIP was performed as described (Mumbach et al., 2016). Briefly, 1 × 10^7^ cells for each HNSCC cell line (1 unique cell line per HNSCC subtype) were crosslinked. *In situ* contacts were generated in isolated and pelleted nuclei by DNA digestion with MboI restriction enzyme, followed by biotinylation of digested DNA fragments with biotin–dATP, dCTP, dGTP, and dTTP. DNA was then sheared with Bioruptor (Diagenode); chromatin immunoprecipitation was done for H3K27Ac with use of anti-H3K27ac antibody. After reverse-crosslinking, 150 ng of eluted DNA was taken for biotin capture with Streptavidin C1 beads followed by transposition with Tn5. In addition, transposed DNA was used for library preparation with Nextera Ad1_noMX, Nextera Ad2.X primers, and Phusion HF 2XPCR master mix. The following PCR program was performed: 72°C for 5 min, 98°C for 1 min, then 11 cycles at 98°C for 15 s, 63°C for 30 s, and 72°C for 1 min. Afterward, libraries were two-sided size selected with AMPure XP beads. Libraries were paired-end sequenced with reading lengths of 76 nucleotides.

Using HiC-Pro (Servant et al., 2015), HiChIP paired-end reads were aligned to the hg19 genome with duplicate reads removed, assigned to MboI restriction fragments, and filtered for valid interactions. Interaction matrices were then generated with the same software. To generate anchor points for downstream looping analysis, outputs from HiC-Pro were used as inputs for peak calling in HiChIP-Peaks (Shi et al., 2020). To ensure loops were called from similar background enhancers, peaks from HiChIP-Peaks were concatenated into a single file and used as anchor point inputs for loop calling via hichipper (Lareau and Aryee, 2018). HiChIP loop visualization was performed using DNAlandscapeR (https://molpath.shinyapps.io/dnalandscaper/).

### Statistical analysis

Statistical analyses, including generation of graphs and plots, were performed using R versions 3.4.4 and 3.6.0. Significance levels are * = *p* < 0.05, ** = *p* < 0.01, and ***p < 0.005 unless otherwise indicated in figure legends. Statistical tests utilized are as indicated in respective text and figure legends.

## Results

### Head and neck squamous cell carcinoma cell lines assigned to known molecular subtypes have similar mutation profiles and tissue origins

**FIGURE 1:**
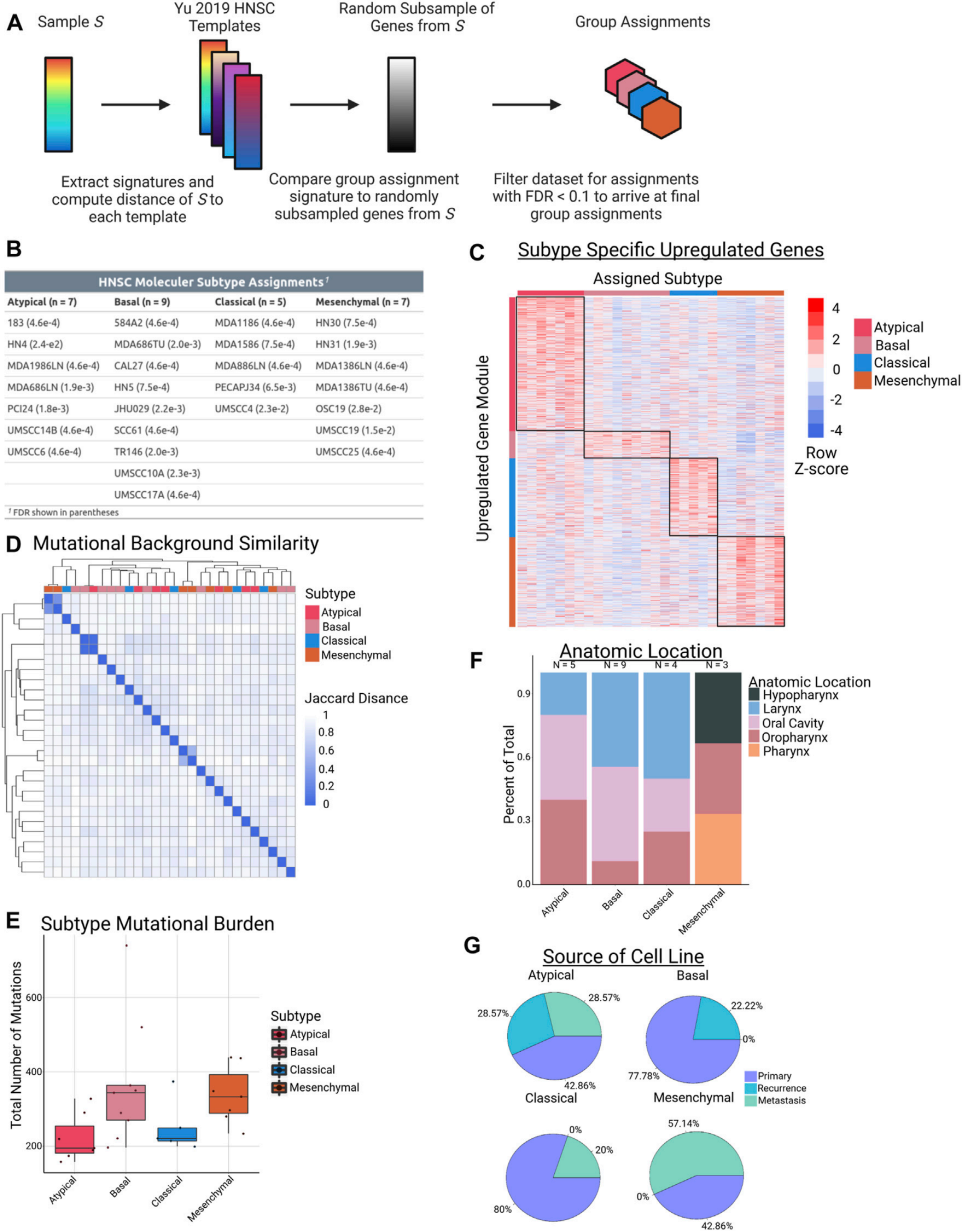
Cell line subtype assignments and characteristics. **(A)** Schematic of workflow used to assign HNSCC cell lines to their respective subtypes using RNA-seq data. **(B)** Table of subtype assignments for each of the 28 cell lines used in this study. **(C)** Heatmap of gene expression modules in each molecular subtype, defined as FC > 3 in a one-vs-rest comparison. **(D)** Hierarchical clustering of the 28 cell lines based on Jaccard distance metrics obtained from binarized mutation counts from WES data. **(E)** Boxplots demonstrating total number of mutations in each sample, grouped by molecular subtype (*p* = NS for each comparison). **(F)** Stacked barplot showing distribution of cell line anatomic location for each molecular subtype. **(G)** Pie chart showing percentage of samples in each molecular subtype that came from primary, recurrent, or metastatic lesions.

Identifying inter-tumor heterogeneity can help better understand the diversity of biological mechanisms driving the neoplastic phenotypes within pathology-based tumor types (e.g., breast cancer, colon cancer, etc.) and discover targeted therapies for specific patient populations (Guinney et al., 2015; Fragomeni et al., 2018; Collisson et al., 2019; Vasaikar et al., 2019). We sought to define heterogeneity within HNSCC patients, especially at the epigenetic level. To this end, we first leveraged published work by Yu et al., which extensively studied CCLE cancer cell lines and their appropriateness as models of human cancer by comparing them to corresponding TCGA tumors (Yu et al., 2019). As part of this work, the group generated “templates” of gene expression values for numerous subtypes in 9 different tumor types. Using the HNSCC templates from this study (one per molecular subtype) and RNA-seq data from the panel of cell lines available to us (Supplementary Table S1), we utilized the nearest template prediction method, as implemented in the CMScaller R package, to assign our cell lines to their most representative molecular subtype (Figure 1A) (Hoshida, 2010; Eide et al., 2017). After selecting only samples with an assignment FDR <0.1, twenty-eight HPV-negative HNSCC cell lines were successfully matched to a molecular subtype, resulting in 7 AT samples, 9 BA samples, 5 CL samples, and 7 MS samples (Figure 1B). Analysis of RNA-seq data in one-versus-rest comparisons demonstrated varying levels of differential expression based on subtype, with a large number of upregulated genes (FC > 3, *n* = 756) in the AT subtype (Figure 1C).

To further investigate a potential genomic basis that could be driving the transcriptomic partitioning into molecular subtypes, we investigated WES data on our panel of cell lines. We first clustered our lines based on their mutational background, and, interestingly, we did not observe any clustering of samples from the same molecular subtype. In fact, the only examples of tight clusters in the data came from 3 matched pairs of cell lines in which the samples were either from a primary or metastatic lesion of the same patient (Figure 1D). Similarly, we did not observe any significant differences between subtypes based on total mutational burden (Figure 1E). This finding is largely consistent with HPV-negative TCGA data, with the singular exception of the AT vs. MS comparison showing a significant difference in mutation number in TCGA (p.adj = 0.031) (Supplementary Figure S1) (Cancer Genome Atlas, 2015). Importantly, observed clustering was neither associated with the anatomic site of origin of the primary tumor from which each the cell lines were derived, nor with the type of tumor the sample was from (e.g., primary vs. recurrence). With the possible exception of the MS subtype, all of the HNSCC subtypes had a fairly equal distribution of samples from the oral cavity, oropharynx, and larynx (Figure 1F). We note this varies from the findings in the TCGA data, where subtype was correlated with anatomic location (Cancer Genome Atlas, 2015). With respect to cell line source, the AT subtype was the only one to contain cell lines from all 3 groups of samples (primary, recurrence, and metastasis), while BA contained only primary and recurrence, and CL and MS contained only primary and metastasis (Figure 1G). Taken together, these results demonstrate that HNSCC molecular subtypes can be successfully assigned to cell lines using RNA-seq data, and that despite their transcriptomic differences, the unique HNSCC subtypes do not have significantly different mutational backgrounds, overall mutation burden, or tissues of origin from one another in our cell line models.

### Head and neck squamous cell carcinoma molecular subtypes are associated with distinct enhancer landscapes

**FIGURE 2:**
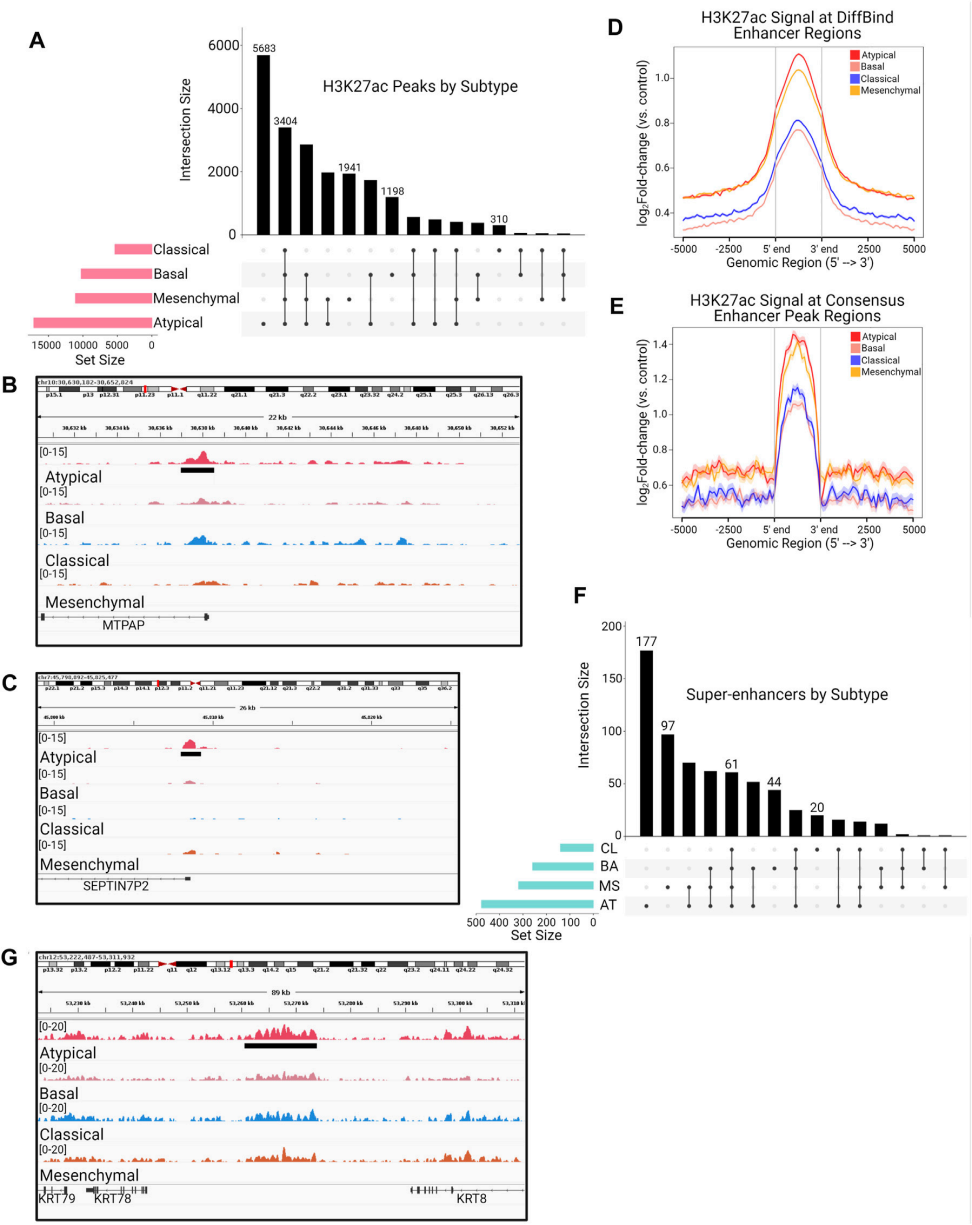
The Atypical subtype is associated with unique enhancer peaks regulating genes related to lipid metabolism and MAPK signaling. **(A)** UpSet plot showing the total number of H3K27ac typical enhancer peaks in each molecular subtype (pink horizontal barplot), as well as the number of peaks in each possible intersection of peaksets (black bars and dot plot). **(B**,**C)** Visualization of mean bigWig signal for each subtype at **(B)** MAP3K8 and **(C)** IGFBP3 enhancer loci containing H3K27ac peaks unique to the AT subtype (green bar/grey shading). **(D**,**E)** H3K27ac ChIP-seq enrichment plots of enhancer loci common to all HNSCC molecular subtypes, defined as **(D)** any peak contained within 2 or more individual samples or **(E)** the 3,404 peaks shared among all consensus peaksets in **(A)**, demonstrating the strongest signal in the AT subtype. **(F)** UpSet plot showing the total number of super enhancer peaks in each molecular subtype (blue horizontal barplot), as well as the number of super enhancer peaks in each possible intersection of peaksets (black bars and dot plot). **(G)** Visualization of mean bigWig signal for each subtype at a MAP3K12 super enhancer containing peaks unique to the AT subtype (green bar/grey shading).

Transcription of a gene is regulated by concerted action of multiple complexes on specific epigenetic elements located in *cis* or *trans* the gene promoter. Enhancers are a major component of the gene regulation circuits and known to be deregulated in cancers (Lee and Young, 2013; Herz et al., 2014). They act as binding platforms for transcription factors that, upon various environmental cues relayed by the cell surface signaling pathways, cooperate with chromatin modifying and remodeling machinery to activate target genes (Lee and Young, 2013). We hypothesized that these transcriptional subtypes could have underlying differences in gene regulatory landscapes that could partly explain the observed transcriptomic differences. To investigate these differences, we generated enhancer profiles for each cell line by performing ChIP-seq for the H3K27ac histone mark, which is widely used as a marker of active enhancers (Creyghton et al., 2010; Rada-Iglesias et al., 2011). We next generated consensus peak sets for each subtype by overlapping the enhancer regions of all cell lines within a subtype and taking the set of enhancers that occurred in 2 or more samples of the subtype. This resulted in 4 total consensus peak sets, each representing a unique subtype (i.e., one consensus peak set per subtype). We observed distinct enhancer peak enrichment among the four molecular subtypes (Figure 2A) (Lex et al., 2014; Khan and Mathelier, 2017). Notably, we discovered the AT subtype has a much larger set of consensus enhancers than any of the other subtypes, and the most common subset of enhancers in our analysis is the set that is unique to the AT subtype (Figure 2A). To investigate the function of the 5,683 enhancers unique to the AT subtype, we utilized data from Cao et al. (Cao et al., 2017), which constructed enhancer-target networks across multiple cancer and sample types, to assign each of these enhancers to their target genes (Figures 2B,C). These genes were then used for pathway enrichment analysis, which revealed enrichment for pathways involved in lipid metabolism, MYC signaling, and MAPK signaling (Figures 2B,C, Supplementary Table S2).

In addition to identifying unique enhancers, we investigated the total H3K27ac signal enrichment across enhancers shared by all 4 subtypes to determine if, in addition to the largest number of H3K27ac peaks, the AT subtype also had greater signal enrichment overall. Indeed, using two separate methods to arrive at a “shared” H3K27ac peak set, we observed that the AT subtype had more enrichment of H3K27ac signal across enhancers shared among all HNSCC subtypes (Figures 2D,E). In agreement with our typical enhancer analysis, we found that the AT subtype also harbored the largest number of called super-enhancers (Figures 2F,G). Further, linking of these super-enhancers to their gene targets not only displayed enrichment for MAPK signaling and lipid metabolism, but also identified enrichment for PI3K and WNT-β-catenin signaling (Supplementary Table S3). These results demonstrate that the AT subtype of HNSCC is enriched for H3K27ac-marked typical enhancers and super-enhancers compared to other HNSCC, and these enhancer regions may activate important cell signaling pathways that are associated with aggressive HNSCCs.

### The Atypical subtype is more resistant to bromodomain inhibition

Recently, the realm of “epigenetic” therapies for cancer has been of major interest for both research and in clinical applications (Castilho et al., 2017; Cheng et al., 2019; Bates, 2020). Targeting epigenetic modifications and the proteins that regulate their placement and/or removal is a particularly attractive approach to cancer therapeutics since these modifications are generally considered to be reversible, particularly when compared to more “permanent” changes such as mutations and copy number alterations. One class of compounds with numerous clinical trials for a variety tumor types is BRD and extraterminal domain (BET) inhibitors, which function by inhibiting the “reader” proteins responsible for recognizing and propagating the signal of acetylated histone residues. These inhibitors have been used in prior studies as enhancer-blocking agents, and the pathways we found to be activated by AT-specific enhancers and super-enhancers (e.g., MAPK and PI3K signaling) are well-characterized mechanisms of BET inhibitor resistance (Supplementary Tables S2, S3) (Rathert et al., 2015; Kurimchak et al., 2016; Iniguez et al., 2018; Cochran et al., 2019; Loganathan et al., 2019; Tonini et al., 2020; Yan et al., 2020). Hence, we hypothesized that the AT subtype may be differentially responsive to BET inhibition.

**FIGURE 3:**
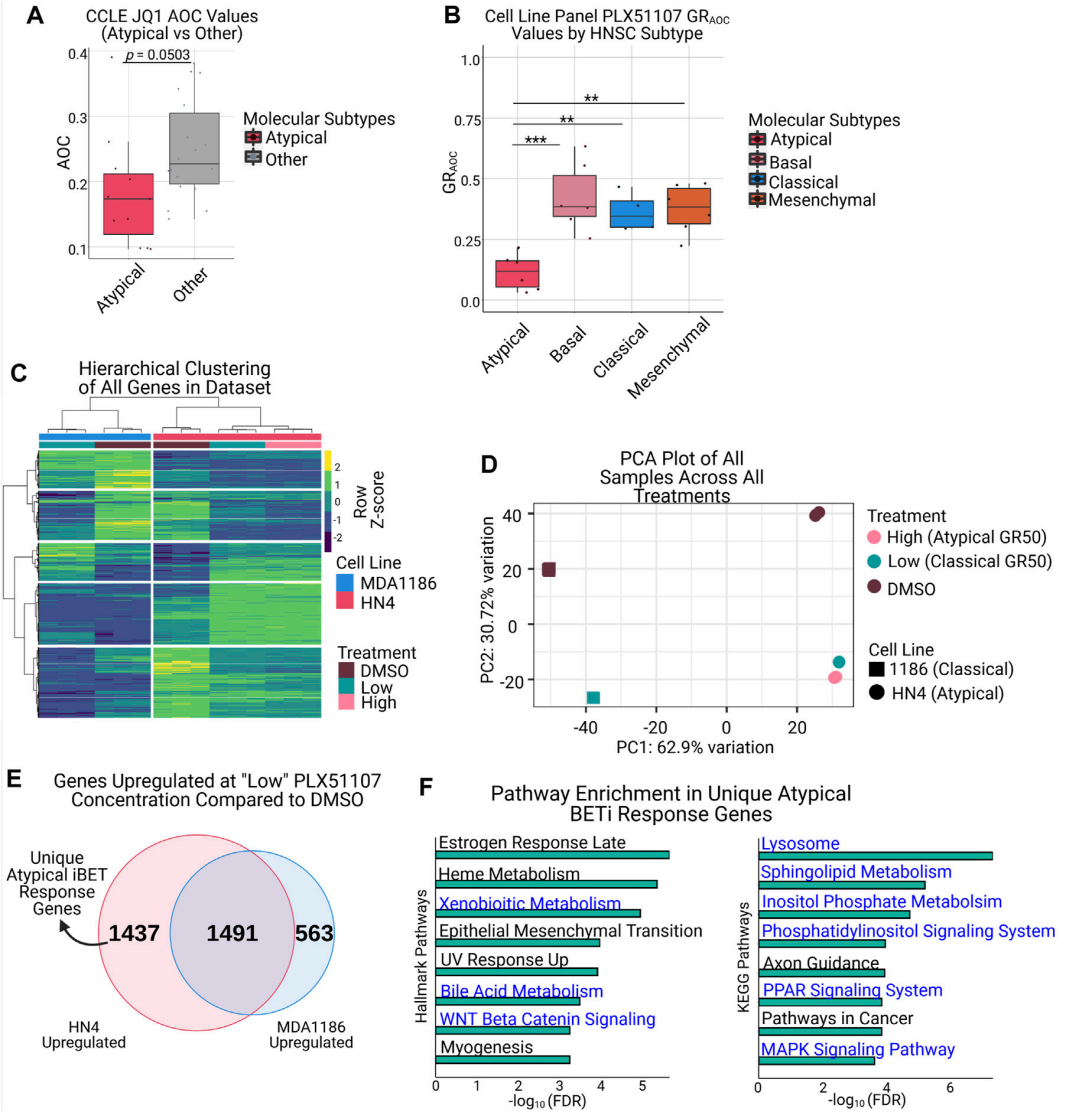
Atypical HNSCC shows increased resistance to BET inhibition and uniquely upregulates genes associated with resistance pathways upon treatment. **(A)** Atypical samples in the HNSCC CCLE dataset demonstrate lower JQ1 AOC values than non-Atypical samples (*p* = 0.0503, Welch’s *t*-test). **(B)** Drug response assays with the BET inhibitor PLX51107 demonstrate the Atypical subtype is significantly more resistant to BET inhibition than other molecular subtypes (*** *adj.p* < 0.001, ** *adj.p* < 0.01). **(C)** Hierarchical clustering of all genes from HN4 and MDA1186 samples treated with DMSO, PLX51107 at GR_50_ MDA1186 (low), or GR_50_ HN4 (high). **(D)** PCA plot of samples as described in **(C)**, displaying separation on the basis of cell line (PC1) and treatment status (PC2). **(E)** Overlap of genes upregulated (|log_2_fold-change| > 1.5 & FDR <0.05) in HN4 and MDA1186 at PLX51107 low concentration; numbers in Venn diagram represent size of set. **(F)** Horizontal barplots of Hallmark (left) and KEGG (right) pathway enrichment results from the 1,437 genes uniquely upregulated by HN4 in **(E)**; pathways highlighted in blue are associated with known mechanisms of BET inhibitor resistance.

We first investigated HNSCC cell line response to JQ1 using the publicly available CCLE drug response data (Basu et al., 2013; Seashore-Ludlow et al., 2015; Rees et al., 2016). We used the available CCLE RNA-seq data to assign samples to their respective HNSCC molecular subtype, then compared their response to BET inhibition in this dataset. Interestingly, we found that the AT samples have a lower JQ1 AOC (Area Over the Curve, where lower values indicate resistance) than those in the non-AT group (*p* = 0.0503, Welch’s *t*-test) (Figure 3A, Supplementary Figure S2A), indicating the AT subtype is more resistant to JQ1 treatment. To extend this analysis, we selected 2 compounds currently being evaluated in clinical trials, OTX015 (Birabresib) and PLX51107, and performed drug response assays in our HNSCC cell lines (Bates, 2020). For each compound, we selected representative cell lines for each molecular subtype (3 AT, 3 BA, 2 CL, and 3 MS), treated with the respective compound for 72 h, then computed the GR_AOC_ of each cell line using cell confluence as a proxy for cell number. We elected to use the GR_AOC_ metric for drug response since GR metrics have been demonstrated to be more reproducible than traditional metrics, such as Area Under the Curve (AUC) and IC_50_, when measuring drug sensitivity in cancer cell lines (Hafner et al., 2016). As we anticipated based on our previous analysis, the BET inhibitor PLX51107 demonstrated significantly lower GR_AOC_ values in the AT subtype compared to any other HNSCC subtype, indicating an increased resistance to treatment in that group (Figure 3B). The inhibitor OTX015 also displayed a similar trend towards increased resistance in the AT subtype that was similar to, but more pronounced than, the JQ1 response data in the CCLE database (Supplementary Figures S2A,B). Given that BRD proteins are responsible for mediating gene transcription and are the main targets of BET inhibitors, we investigated the expression of this family of proteins both in our cell line panel and in the HNSCC TCGA data (Rathert et al., 2015; Kurimchak et al., 2016; Iniguez et al., 2018; Cheng et al., 2019; Cochran et al., 2019). This analysis demonstrated a significant elevation in BRD7 in the AT subtype compared to other subtypes in our HNSCC cell lines (Supplementary Figures S2C,D), and a significant elevation in BRD4 expression in AT vs. BA and AT vs. MS comparisons in the HNSCC TCGA data (Supplementary Figures S2E,F).

To better understand the mechanisms behind this observed resistance to treatment, we selected one representative cell line from the AT subtype (HN4) and one cell line from the non-AT subtypes (MDA1186, CL subtype), treated with PLX51107 or DMSO, and performed gene expression analysis using mRNA-seq profiling in each condition. MDA1186 was treated with PLX51107 at its own GR_50_ value (hereafter referred to as “low” concentration), and HN4 was treated at its own GR_50_ value (hereafter referred to as “high”), as well as the low concentration. To ensure PLX51107 behaved similarly to other published BET inhibitors, we created BET inhibitor response signatures using publicly available data and, using GSEA, confirmed that the response of the AT and non-AT cell lines to PLX51107 was consistent with previously documented BET inhibitor response signatures (Supplementary Figures S3C,D) (Puissant et al., 2013; Picaud et al., 2016). Hierarchical clustering of all genes in the RNA-seq dataset demonstrated 2 major clusters, one per cell line, as well as 2 sub-clusters, either DMSO or PLX51107-treated, per major cluster (Figure 3C). To further examine the differences in response to drug treatment, we performed a principal component analysis (PCA), which revealed a first component driven by the cell line identity, and a second component driven by treatment status (Figure 3D). Examining the results from the hierarchical clustering and PCA analysis together, we noted that the majority of transcriptional response to BET inhibition in the AT cell line occurs at the lower drug concentration, and only a minority of gene expression changes occur between the low and high concentrations (Figures 3C,D). Closer inspection of the gene expression heatmap indicates that the genes that are specifically upregulated in the AT subtype after PLX51107 treatment, but not in the non-AT subtypes, may be responsible for mediating resistance to BET inhibition (Figure 3C).

To investigate these uniquely upregulated genes, we overlapped the set of genes upregulated by the AT subtype and by the non-AT subtype at the low PLX51107 concentration. As we expected, we discovered a set of 1437 genes uniquely upregulated in the AT subtype after BET inhibition (Figures 3C,E). Pathway analysis of these 1437 genes using the Hallmarks and KEGG gene sets reveals enrichment for multiple pathways previously demonstrated to convey resistance to BET inhibition and identified by our previous enhancer-based analysis, including MAPK signaling, WNT-β-catenin signaling, phosphatidylinositol signaling, and lipid metabolism pathways (Figure 3F) (Rathert et al., 2015; Kurimchak et al., 2016; Iniguez et al., 2018; Cochran et al., 2019; Loganathan et al., 2019; Tonini et al., 2020; Yan et al., 2020).

These results support our previous hypothesis that the AT subtype is more resistant to BET inhibition than other HNSCC subtypes and suggest the enrichment of H3K27ac-marked enhancers involved in these pathways is a contributing factor to the observed resistance.

### Bromodomain inhibitor resistance is mediated by baseline enhancer activity and chromatin structure

Our previous analyses have indicated that the AT subtype of HNSCC has two intriguing properties with respect to BET inhibition: first, H3K27ac-marked enhancers unique to the AT subtype regulate genes enriched for known BET inhibitor resistance pathways, and, second, the AT subtype is able to uniquely upregulate genes enriched for BET inhibitor resistance pathways after treatment with BET inhibitor (Figures 2B,C,G; Supplementary Tables S2, S3; Figures 3E,F). Because of these observations, we suspected the AT subtype may have a stronger baseline enhancer activity at genes involved in resistance pathways and that these genes may have higher numbers of enhancers-promoter contacts, enabling a more robust response to BET inhibitor treatment.

**FIGURE 4:**
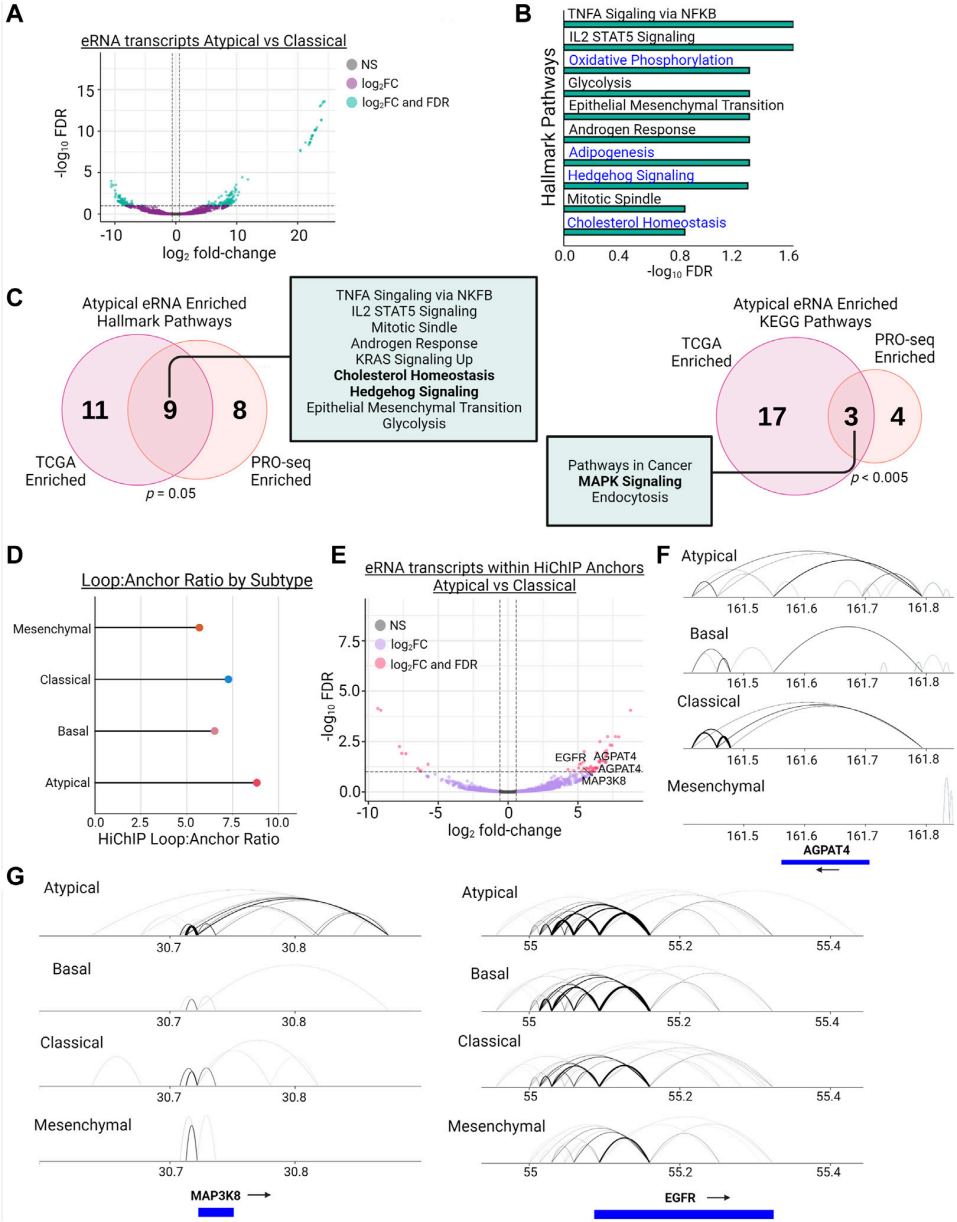
Enhancers of MAPK signaling, WNT signaling, and Cholesterol Homeostasis genes display increased eRNA transcription and enhancer-promoter looping in Atypical HNSCC. **(A)** Differential transcription (|log_2_fold-change| > 1.5 & FDR <0.1) of eRNAs between the Atypical and Classical subtypes as measured by PRO-seq (green dots meet fold-change and FDR thresholds, purple dots meet fold-change threshold only). **(B)** Hallmark pathway enrichment analysis of genes linked to eRNAs with significantly increased transcription from **(A)**; pathways in blue have been previously associated with BET inhibitor resistance. **(C)** Overlap of hallmark (left) and KEGG (right) pathway enrichment results between PRO-seq-determined significantly enriched eRNAs from **(A)** and **(B)** and TCGA-measured differentially expressed eRNAs between Atypical and non-Atypical samples; *p* values represent hypergeometric tests of gene set enrichment result overlaps; bolded terms represent shared pathways associated with BET inhibitor resistance. **(D)** Lollipop plot demonstrating the loop count:anchor count ratio of H3K27ac HiChIP data for each molecular subtype. **(E)** Volcano plot of differentially transcribed (|log_2_fold-change| > 1.5 & FDR <0.1) eRNAs between the Atypical and Classical subtypes after filtering transcripts for only those contained within H3K27ac HiChIP anchors (pink dots meet fold-change and FDR thresholds, purple dots meet fold-change threshold only). **(F)** Joined Hallmark and KEGG pathway enrichment analysis of genes linked to differentially transcribed eRNAs in **(E)**; pathways in blue have been previously associated with BET inhibitor resistance. **(G)** Visualization of H3K27ac HiChIP loops at the MAP3K8 locus (left) and EGFR locus (right) in all 4 HNSCC molecular subtypes.

To investigate the activity of enhancers involved in regulating baseline resistance gene expression, we performed PRO-seq to investigate the eRNA landscape of the AT and non-AT subtypes (Supplementary Table S4) (Mahat et al., 2016). Enhancer RNAs are a recently discovered class of non-coding transcripts found at active enhancers that arise from the transcription of enhancer elements themselves and are involved in functions such as regulating gene transcription and controlling enhancer-promoter looping (Arnold et al., 2019; Zhang et al., 2019; Sartorelli and Lauberth, 2020). We used PRO-seq, with a particular focus on eRNAs, to investigate differential enhancer activity in our AT subtype. For this experiment, we expanded our AT group to include the 3 cell lines from our drug assay, and we expanded the non-AT group to include the 2 cell lines from the CL subtype used in our drug assay. Differential expression analysis of PRO-seq-defined eRNAs revealed 321 differentially expressed eRNAs, with 207 being upregulated and 114 being downregulated (Figure 4A).

To assess the likely functional output of these eRNAs, we assigned each eRNA to its nearest gene and performed pathway enrichment analysis, which demonstrated an enrichment in multiple metabolic pathways, including lipid metabolism and cholesterol homeostasis, and hedgehog signaling (Figure 4B). These findings are largely in agreement with our previous enhancer-based analysis of H3K27ac-linked genes, which displayed enrichment for similar BET inhibitor resistance associated pathways (Supplementary Table S2). To extend this finding to human tumors, we leveraged data from recent publications investigating eRNA expression in TCGA tumors (Cheng et al., 2019; Chen and Liang, 2020). After assigning all the HNSCC TCGA tumor samples to their molecular subtype, we examined eRNA expression in the AT subtype compared to non-AT samples and found the AT subtype upregulated 1,867 eRNAs. After linking these eRNAs to their nearest gene, we found that, in agreement with our PRO-seq data from HNSCC cell lines, these genes were enriched for cholesterol homeostasis, hedgehog signaling, and MAPK signaling function (Figure 4C). Next, we overlapped both our HNSCC cell line PRO-seq data and the TCGA HNSCC eRNA data with the predicted enhancers for Cao et al. (Cao et al., 2017) and found that 52% (3766/7246) of the cell line PRO-seq enhancers and 67% of the TCGA eRNA enhancers (42391/63479) matched a predicted enhancer (Supplementary Figures S4). These results support the hypothesis that the AT subtype has active enhancers, as measured by eRNA expression, enriched for signaling pathways that have been demonstrated to confer resistance to BET inhibition across cancer types.

To assess the enhancer-promoter contacts in our HNSCC cell lines, we performed HiChIP for H3K27ac-marked histones to capture the E-P looping involving this enhancer mark (Supplementary Figure S5) (Mumbach et al., 2016). Consistent with the previous enhancer analyses, we discovered that the AT subtype has the highest ratio of H3K27ac-mediated loops to H3K27ac anchors across all 4 HNSCC subtypes, indicating the AT subtype may have more redundancy in its enhancer architecture than the other subtypes (Figure 4D). To assess if these loops are related to enhancer function, we overlapped our PRO-seq called eRNA enhancer regions with the H3K27ac HiChIP anchor data and performed differential expression analysis of this subset of eRNAs. We identified 48 of 57 differentially transcribed eRNAs as upregulated, and these eRNAs were associated with genes involved in MAPK signaling and lipid metabolism, such as MAP3K8, EGFR, and AGPAT4 (Figure 4E). Inspection of genes identified by this integrative analysis revealed increased contact formation between respective gene promoters and H3K27ac-marked enhancers, supporting the association of eRNA expression with active enhancers and enhancer-promoter loop formation (Figures 4F,G). Further, we compared our HiChIP data for MAP3K8, EGFR, and AGPAT4 to the Cao et al. (Cao et al., 2017) predicted enhancers, which, while demonstrating variable numbers of overlaps depending on the gene queried, maintained the enrichment of enhancer looping in the AT subtype (Supplementary Tables S5, S6). Increasing the loop call stringency by increasing the number of required paired-end tags (PETs) further exaggerated the enrichment of E-P looping in the AT subtype (Supplementary Table S6).

Overall, insights from the eRNA expression and HiChIP data support a model in which the AT subtype has more active enhancers regulating genes associated with lipid metabolism and MAPK signaling, and AT enhancers have, on average, a higher level of redundancy in their control of gene expression than non-AT enhancers by forming larger numbers of enhancer-promoter contacts.

## Discussion

Here, we have demonstrated that HNSCC cell line molecular subtypes have largely similar mutational backgrounds, mutational burden, and anatomic sites of origin. In contrast, the enhancer landscapes, marked by histone H3K27 acetylation, are distinct among subtypes. In particular, we discovered the AT subtype has the highest number of enhancers and super-enhancers, as well as the most enhancer signal at common enhancer regions and a global increase in enhancer-promoter loop formation. We also demonstrate that the AT subtype is more resistant to BET inhibition and that, upon treatment with BET inhibitors, the AT subtype is able to uniquely upregulate genes associated with cell growth and BET inhibitor resistance pathways (MAPK signaling, WNT signaling, and lipid metabolism) (Rathert et al., 2015; Kurimchak et al., 2016; Iniguez et al., 2018; Cochran et al., 2019; Loganathan et al., 2019; Tonini et al., 2020; Yan et al., 2020). Further, we demonstrate a significant baseline upregulation of eRNA transcription from the enhancers of genes involved in BET inhibitor resistance pathways such as lipid metabolism and hedgehog signaling in the AT subtype (Cochran et al., 2019). Interestingly, many of these genes with increased eRNA expression in their enhancers were also found to have baseline increased enhancer-promoter looping. Together, our findings suggest that the AT subtype of HNSCC is characterized by high enhancer activity, which likely drives the expression of pathways known to confer resistance to BET inhibition.

Delineation of HNSCC into 4 subtypes was originally proposed by Walter et al. and the TCGA HNSCCC study (Walter et al., 2013; Perez Sayans et al., 2019). These two manuscripts largely focus on genomic alterations, such as copy number alterations and somatic mutations, and only one epigenetic element, in the form of DNA methylation, was assessed in the TCGA paper. As such, limited epigenomic data for HNSCC is available (Serafini et al., 2020). Despite these limitations, interest in therapies that target the epigenome continues to grow, indicating a need for more studies that focus on the epigenome of HNSCC (Alsahafi et al., 2019; Bates, 2020). The work presented here is, to our knowledge, the first to characterize the enhancer landscape of HNSCC based on the Walter/TCGA molecular subtypes. Interestingly, we identified HPV-negative samples belonging to AT subtype, which has traditionally been associated with HPV-positive or “HPV-like” samples, have increased enhancer activity compared to the non-AT subtypes. This activity is measured by increased H3K27ac peak counts, increased H3K27ac signal at common enhancer peaks, global increases in enhancer-promoter looping, and significant upregulation of eRNA expression compared to non-AT samples. This finding suggests that defining features of the AT subtype are enhancer architecture and activity - two key epigenomic aspects of HNSCC subtypes that were not previously explored. Clinical and translational significance of enhancer-based classification was shown by our recent studies in other tumor types like colorectal cancer (Orouji et al., 2021) and MPNST (Kochat et al., 2021).

Unfortunately, BET inhibitors have shown limited promise in clinical studies in solid tumors (Shorstova et al., 2021). However, in specific solid tumor contexts, such as BRD4-NUT midline carcinoma, BET inhibitors have had very encouraging results in clinical trials (Stathis et al., 2016; Piha-Paul et al., 2020; Shorstova et al., 2021). Considering these findings, identifying subsets of patients with tumor biology favorable or unfavorable to BET inhibitor response could improve the clinical utility of these compounds. Since BET inhibitors inherently rely on modulating the reader protein of H3K27ac-marked enhancers in target cells, understanding enhancer landscapes and their role in BET inhibitor response becomes an important first step in sorting patients into “favorable” or “unfavorable” groups (Stathis and Bertoni, 2018; Cheng et al., 2019). In our work, we discovered that the AT subtype is significantly more resistant to BET inhibition than other HNSCC subtypes, and that this resistance seems, at least in part, mediated by increased enhancer activity on pathways associated with lipid and cholesterol metabolism, MAPK signaling, and WNT-β-​catenin signaling. Accordingly, we expect that including compounds that target these pathways in combination with BET inhibitors may sensitize otherwise resistant tumors to BET inhibition and expand the current chemotherapeutic repertoire for HNSCC treatment. As such, other enhancer/transcription blocking inhibitors, such as those against CDK9 (Zhang et al., 2018), could be tested in such enhancer-based subtypes.

We acknowledge that a limitation of our work is the focus on cell line models of HNSCC, which has certain limitations compared to studying human tumors directly. In particular, we noticed differences in BRD expression patterns between our cell line RNA-seq data and the HNSCC TCGA dataset, and the possibility of this being at least partially the result of the sample sources cannot be excluded and warrants further investigation in subsequent studies. However, given the relative sparsity of data in the HNSCC enhancer regulation space, our data can serve as a valuable resource as this field continues to grow. The work presented in this manuscript also serves as an early investigation into the enhancer regulatory landscape of HNSCC using multiple methods that can be technically challenging to perform in human tissue because of the amount of sample required and the associated difficulty of acquiring sufficient numbers of human samples. Moving forward, it will be important to perform similar studies in human tumor samples and animal models to corroborate the findings from our work in an *in vivo* setting.

While our work focused on HPV-negative HNSCC, our findings suggest increased enhancer activity on genes involved in lipid and cholesterol metabolism, MAPK signaling, and WNT-β-​catenin signaling may serve as a general mechanism of baseline resistance to BET inhibition. Since enhancer architecture is a critical component of cell identity, it is possible that, moving forward, an assessment of a tumor’s baseline enhancer activity could serve as a potential epigenomic biomarker of response to BET inhibition and aid in tailoring treatment in a patient-specific manner (Hnisz et al., 2013; Kron et al., 2014). This could be especially useful in the case of HNSCC, where subtype-specific and tumor-specific treatments are generally lacking (Alsahafi et al., 2019).

## Data Availability

Processed data is available from the Gene Expression Omnibus under repositories GSE185531 (ChIP-seq), GSE185532 (HiChIP), GSE185533 (PRO-seq), and GSE185534 (mRNA-seq). Please note that the raw sequence data cannot be provided due to these being old cell lines without proper consents. Please contact KR for the raw sequence data. Relevant code for the manuscript can be found at: https://gitlab.com/railab/hnscc_subtypes.

## Supplementary material

Supplementary Figure S1 Boxplots demonstrating total number of mutations in each sample, grouped by molecular subtype (*p.adj* = .031 for AT vs. MS, *p.adj* = NS for all other comparisons).

Supplementary Figure S2
**(A)** CCLE-derived JQ1 AOC values grouped by individual HNSC molecular subtype. **(B)** Response of HNSC cell lines to the BET inhibitor OTX015, reported as GR_AOC_ to adjust for cell line growth rates and grouped by molecular subtype (** *adj.p* < 0.01). **(C)** Box and whisker plot of BRD4 expression in HNSCC cell lines (* = *p.adj* < 0.05, NS = not significant). **(D)** Box and whisker plot of BRD7 expression in HNSCC cell lines (* = *p.adj* < 0.05, ** = *p.adj* < 0.01, and *** *p.adj* < 0.005). **(E)** Box and whisker plots of BRD gene expression in TCGA HNSCC samples. **(F)** Box and whisker plot of BRD4 expression in HNSCC cell lines (* = *p.adj* < 0.05, NS = not significant).

Supplementary Figure S3 GSEA analysis of **(A)** MDA1186 and **(B)** HN4 response signatures to 24 h of PLX51107 treatment at their respective GR_50_ values; gene sets for enrichment calculations were generated from previously published BET inhibitor response signatures.

Supplementary Figure S4
**(A)** Venn diagram of overlaps between HNSCC cell line PRO-seq eRNA-based enhancers (orange) and Cao et al. (Cao et al., 2017) predicted enhancers (blue). **(B)** Venn diagram TCGA HNSCC eRNA-based enhancers (magenta), and Cao et al. (Cao et al., 2017) predicted enhancers (blue).

Supplementary Figure S5
**(A**–**D)** HiC-Pro valid interaction and contact range metrics for HN4, CAL27, MDA1186, and UMSCC25 HNSCC cell lines, respectively. **(E)** Number of intrachromosomal paired-end tags (PETs) and distribution in distance bins per sample as reported by hichipper.

Supplementary Table S1 List of cell lines used for H3K27ac ChIP-seq, their total H3K27ac peak counts, and their total uniquely mapped read counts.

Supplementary Table S2 List of top 20 enriched Hallmark and KEGG pathways and their respective FDRs from genes lists generated by linking AT-unique typical enhancers to their target genes.

Supplementary Table S3 List of top 20 enriched Hallmark and KEGG pathways and their respective FDRs from genes lists generated by linking AT-unique super enhancers (sheet 2) to their target genes.

Supplementary Table S4 PRO-seq quality metrics for each HNSCC cell line sample as reported by the PEPPRO pipeline.

Supplementary Table S5 Table showing relationship between Cao et al. (Cao et al., 2017) predicted enhancers and HiChIP anchors for AGPAT4, MAP3K8, and EGFR.

Supplementary Table S6 Table showing relationship between Cao et al. (Cao et al., 2017) predicted enhancers and HiChIP H3K27ac-based loops. The “threshold” value is the number of required PET interactions used for generating Figures 4F,G (10 for AGPAT4, 5 for MAP3K8 and EGFR).
