# Supplementary figures and images for: High enhancer activity is an epigenetic feature of HPV negative atypical head and neck squamous cell carcinoma
Source: Front Cell Dev Biol. 2022 Jul 19;10:936168. doi: 10.3389/fcell.2022.936168 (PMC9343809; doi:10.3389/fcell.2022.936168)

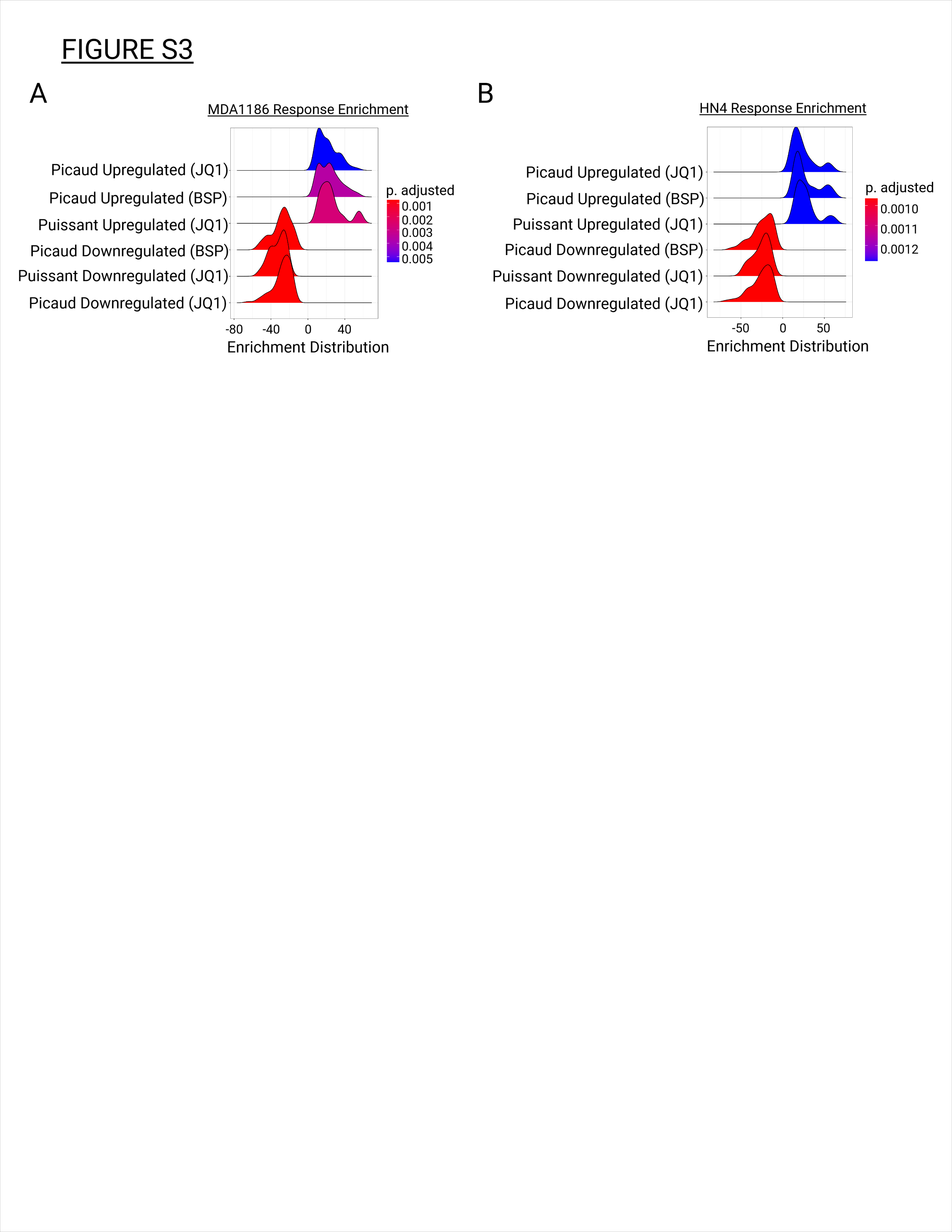

Supplement: Supplementary file 1 [file Image3.JPEG]

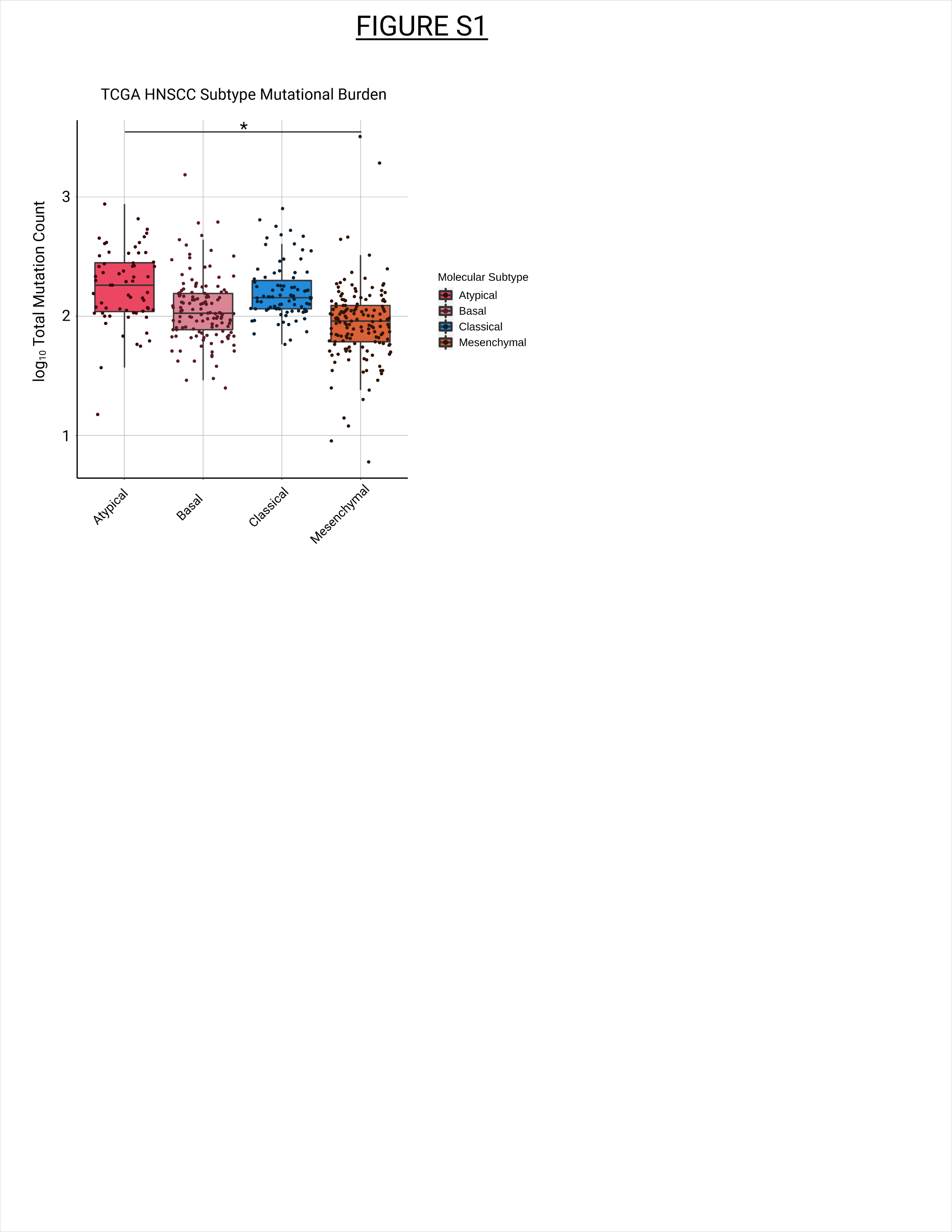

Supplement: Supplementary file 3 [file Image1.JPEG]

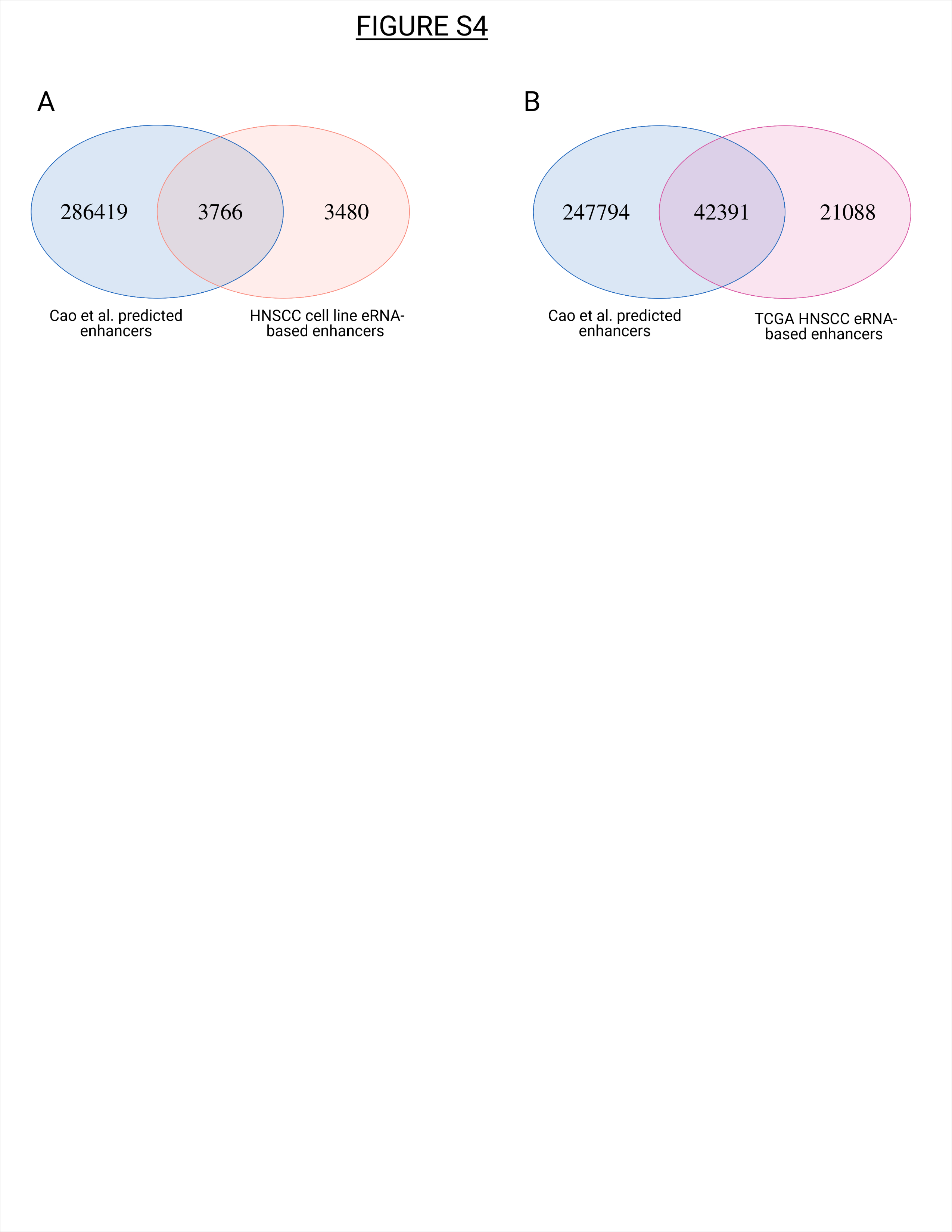

Supplement: Supplementary file 4 [file Image4.JPEG]

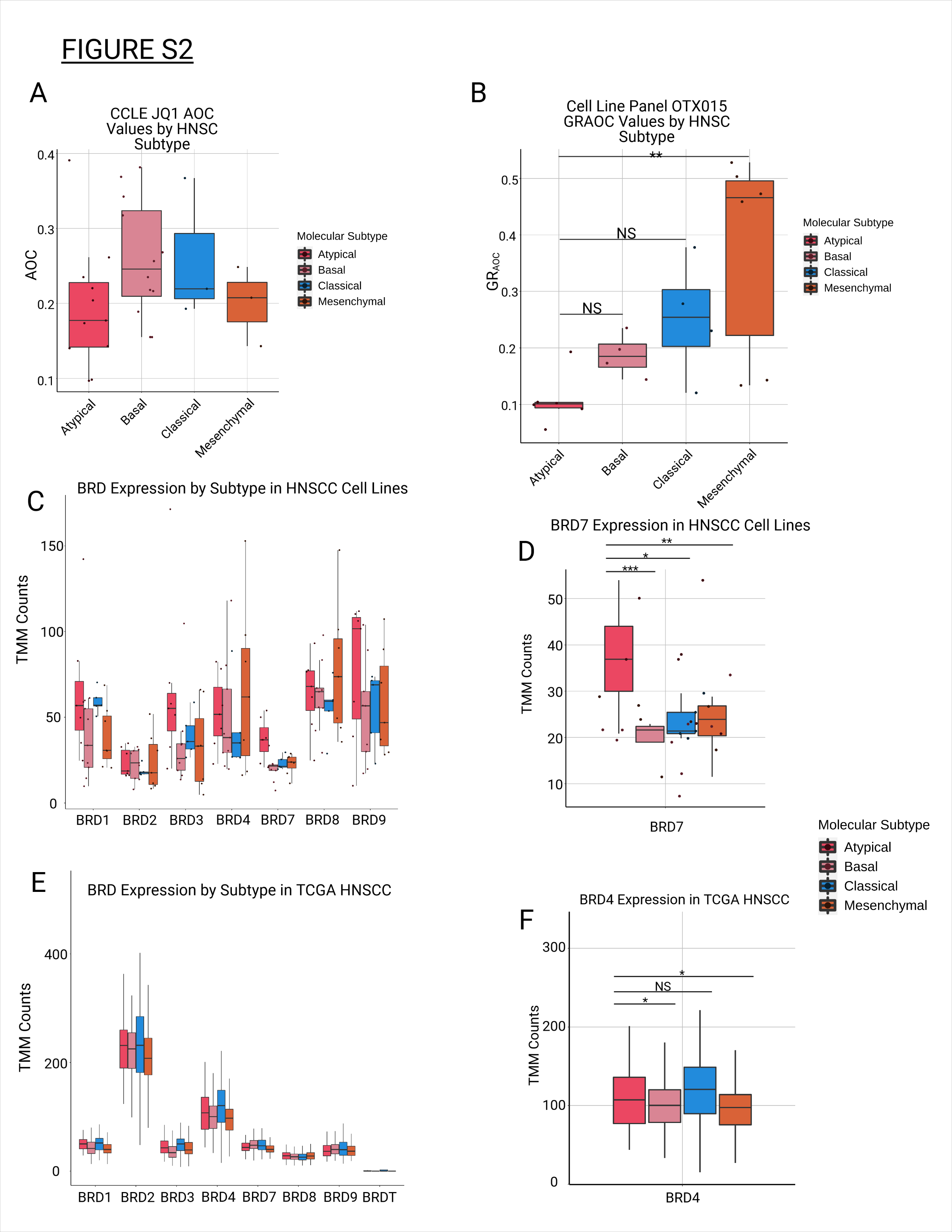

Supplement: Supplementary file 5 [file Image2.JPEG]

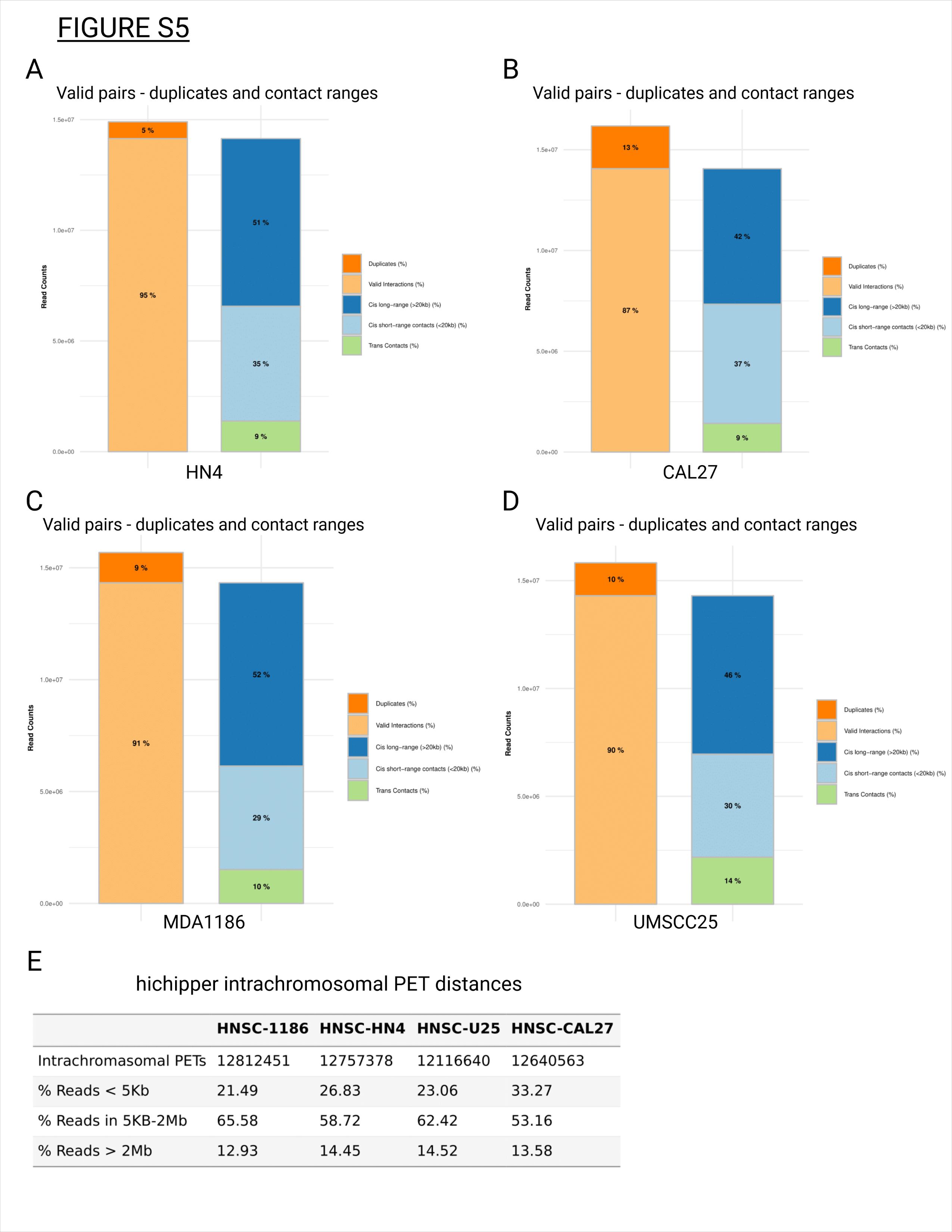

Supplement: Supplementary file 6 [file Image5.JPEG]
